# Parents’ drinking, childhood hangover? Parental alcohol use, subjective health complaints and perceived stress among Swedish adolescents aged 10–18 years

**DOI:** 10.1186/s12889-023-15097-w

**Published:** 2023-01-24

**Authors:** Joakim Wahlström, Charlotta Magnusson, Sara Brolin Låftman, Johan Svensson

**Affiliations:** 1 Department of Public Health Sciences, Stockholm University, SE-106 91 Stockholm, Sweden; 2 Swedish Institute for Social Research, Stockholm University, Stockholm, Sweden

**Keywords:** Parental drinking, Parental alcohol use, Youth, Psychological complaints, Somatic complaints, Stress

## Abstract

**Background:**

Alcohol abuse is not only harmful to the consumer but may also negatively impact individuals in the drinker’s social environment. Alcohol’s harm to others is vital to consider when calculating the true societal cost of alcohol use. Children of parents who have alcohol use disorder tend to have an elevated risk of negative outcomes regarding, e.g., health, education, and social relationships. Research on the general youth population has established a link between parental drinking and offspring alcohol use. However, there is a lack of knowledge regarding other outcomes, such as health. The current study aimed to investigate the associations between parental drinking and children’s psychological and somatic complaints, and perceived stress.

**Methods:**

Data were derived from a nationally representative sample, obtained from the 2010 Swedish Level-of-Living survey (LNU). Parents and adolescents (ages 10–18) living in the same households were interviewed independently. The final study sample included 909 adolescents from 629 households. The three outcomes, psychological and somatic complaints and perceived stress, were derived from adolescents’ self-reports. Parents’ self-reports of alcohol use, both frequency and quantity, were used to categorise adolescents as having abstaining, low-consuming, moderate-drinking, or heavy-drinking parents. Control variables included adolescents’ gender, age, family structure, and household socioeconomic status. Linear and binary logistic regression analyses were performed.

**Results:**

Parental heavy drinking was more common among adolescents living in more socioeconomically advantaged households and among adolescents living with two custodial parents or in reconstituted families. Adolescents with heavy-drinking parents reported higher levels of psychological and somatic complaints and had an increased likelihood of reporting stress, compared with those having moderate-drinking parents. These associations remained statistically significant when adjusting for all control variables.

**Conclusion:**

The current study’s results show that parental alcohol consumption is associated with poorer offspring adolescent health. Public health policies that aim to reduce parental drinking or provide support to these adolescents may be beneficial. Further studies investigating the health-related outcomes among young people living with heavy-drinking parents in the general population are needed to gain more knowledge about these individuals and to implement adequate public health measures.

**Supplementary Information:**

The online version contains supplementary material available at 10.1186/s12889-023-15097-w.

## Introduction

Alcohol use is a leading risk factor for the global burden of disease and entails a substantial economic cost, both directly and indirectly, to many societies [1, 2]. The harmful effects of alcohol consumption also reach beyond the individual and affect the drinker’s social environment. This type of social harm should be considered by researchers and policy makers alike to not underestimate the detrimental societal effects attached to alcohol use [3]. Recently, research into alcohol’s harms to others has grown and covers a broad range of topics, e.g., traffic accidents, violence, mental health problems, and work-related difficulties [4].

Young people are especially vulnerable to others’ drinking and much research has focused on children of parents with alcohol use disorder. The existing literature reveals that these individuals fare worse than other offspring in important life areas such as health, education, and social relationships [5]. Health adversities among youth with parents who have alcohol use disorder encompass both externalising problems, e.g., attention difficulties, lack of impulse control, and aggression, and internalising problems, e.g., depression, anxiety, and low self-esteem [5–7]. Parental alcoholism can be regarded as a stressor in the home environment, which may cause deteriorating family relationships and lead to health issues among offspring [8].

Studies looking beyond clinical samples have found a clear and consistent association between parental alcohol use and adolescent drinking in the general youth population [9]. However, knowledge about other outcomes is lacking [10]. While research from several countries confirms the associations between parental problematic drinking and adverse offspring health among the general population [11–14], prior studies are often limited by their assessment of the parental (problematic) alcohol use measure. Information collected only from the adolescents themselves, in conjunction with a focus on *problematic* drinking, rather than overall consumption, are methodological features that might underestimate the total harms that parental drinking entails since adolescents are not likely to have full knowledge about their parents’ alcohol habits and the parent-child relationship could influence the perception of their parents’ drinking. One exception is a study done on Korean data, which found that parent-reported frequency of drinking was associated with adolescent self-perception of stress [15]. Nonetheless, calls for more studies on alcohol’s harm to others using data collected from the drinker and the victim separately have been made [3]. Examining the links between parental drinking and stress-related outcomes in the offspring seems especially relevant. Previous research has indicated that subjective health complaints reflect stress among adolescents [16]. Subjective health complaints are usually divided into somatic (e.g. headache, stomach ache) and psychological (e.g. nervousness, feeling irritated) symptoms [17], and indicate health difficulties that cannot be attributed to a medical or psychological diagnosis [18, 19]. Recurring complaints may also persist into (early) adulthood and could develop into other, more severe health issues [20, 21].

This study aims to examine the associations between parents’ self-reported quantity and frequency of alcohol consumption and offspring psychological and somatic complaints, and perceived stress, using data collected from parent(s) and adolescents separately.

## Methods

### Data material

The Data were obtained from the 2010 Swedish Level-of Living survey (LNU). LNU is based on a nationally representative sample of 0.1% of the population aged 18–75 and is performed approximately every 10 years, with the 2010 dataset being the most recently available [22]. Personal interviews performed in the respondent’s household are used to gather the data. The survey contains questions on a broad range of living conditions, including, e.g., sociodemographic characteristics, educational and occupational careers, and health. Starting with LNU 2000, two complementary surveys with other family members are also performed, namely Partner-LNU and Child-LNU. The Partner-LNU is offered to the spouses/partners of the main respondent and consists of a shortened version of the main survey. The Child-LNU is offered to adolescents aged 10–18 years who live in the main respondent’s household. Ethical approval has been obtained from the Regional Ethical Review Board of Stockholm (ref. no. 2009/1802-31/5).

For the current study, we combined data from the LNU, Partner-LNU, and Child-LNU of 2010. As data were gathered from the co-habiting partner of the main responding parent, variables based on parental information can include answers from both custodial and/or step-parents. The LNU 2010 survey included 7,253 individuals in the sampling frame and interviews were conducted with 4,415 of these, corresponding to a response rate of 60.9%. Furthermore, 3,347 spouses or partners to the main respondent were identified and 2,522 of these completed the Partner-LNU 2010 (75.4%). In total, 1,238 adolescents were offered to participate in the Child-LNU 2010 and 923 agreed to take part (71.9%). After further exclusion due to internal non-response on any of the variables used in the current study, the analytical sample consisted of 909 adolescents in 629 households. Among these, 640 adolescents (70.4%) had linked information from two parents. Among those living with two custodial parents (n = 634), 83.4% had linked information from both parents.

### Measures

The dependent variables in this study, psychological complaints, somatic complaints, and perceived stress, were based on adolescents’ self-reports from the Child-LNU.

Psychological complaints were constructed from three items: ‘I often feel sad or down’; ‘I am often tense or nervous’; ‘I am often grouchy or irritated’. Response alternatives were: (1) ‘Matches exactly’, (2) ‘Matches roughly’, (3) ‘Matches poorly’, and (4) ‘Does not match at all’. Responses were then reversed and added to an index ranging from 3 to 12, with higher values indicating more psychological complaints (Cronbach’s alpha 0.65). Missing answers on at most one of the items were replaced with the individual mean of the remaining items.

Somatic complaints were constructed from three items: ‘In the last 6 months, how often have you had the following complaints?’: ‘Headache’, ‘Stomach-ache’, ‘Difficulties in falling asleep’. Respondents were given five response alternatives: (1) ‘Every day’, (2) ‘Several times a week’, (3) ‘Once a week’, (4) ‘About once a month’, and (5) ‘More seldom or never’. Responses were then reversed and added to an index ranging from 3 to 15, with higher values indicating more frequent somatic complaints (Cronbach’s alpha 0.55). Missing answers on at most one of the items were replaced with the individual mean of the remaining items. These measures of psychological and somatic complaints have been used previously [23].

Perceived stress was captured by the question: ‘During the last 6 months, how often have you felt stressed?’. Response alternatives were the same as for somatic complaints. Adolescents who felt stressed more than once a week were categorised as reporting perceived stress [24].

The independent variable, parental alcohol use, was based on parent-reported information and constructed from three items in the LNU and Partner-LNU. Parents were categorised as: abstainers; low consumers; moderate drinkers; or heavy drinkers. The first question was: ‘Do you at any time drink wine, strong beer/cider, or liquor?’ and those who answered ‘Yes’ responded to two additional questions: ‘During the last 12 months, about how often have you consumed some amount of alcoholic beverage, that is: wine, strong beer, strong cider or liquor?’ (with response alternatives: ‘Daily or almost daily’, ‘2–4 times a week’, ‘Once a week’, ‘2–3 times a month’, ‘Once a month’, ‘6–11 times a year’, ‘Less often’, and ‘never’) and ‘On such occasions, how many glasses do you usually drink? One glass can be 1 glass of wine, 1 bottle or can of beer, or 1 schnapps or drink’. Non-drinking parents were categorised as abstainers and those who drank less than once a month as low consumers. We wanted to keep these types of drinking patterns separate since current non-drinking or low consumption may be a result of prior heavy consumption and/or indicative of characteristics that may influence offspring health, such as severe health problems among parents or small social networks [25, 26]. Parents who drank daily, regardless of quantity, *or* 2–4 times a week and at least 3 glasses per occasion, were categorised as heavy drinkers. Using this cut-off, parents in the heavy-drinking category consume at least 6 glasses per week. While this figure is well below the Swedish guidelines for what counts as heavy drinking it is important to acknowledge that the response categories given contain variation that is impossible to avoid in the construction of the categories. One individual who answers ‘2–4 times a week’ could drink twice as often as another individual who ticks the same box, for example. The answers that respondents provide are also likely affected by recall bias due to difficulties in remembering how often and how much one drinks, the propensity to underestimate one’s consumption of alcohol because of social desirability, and the hard task of calculating an average number of drinking occasions and drinks for the last 12 months. The difficulty of accurately measuring individuals’ alcohol consumption in epidemiology is known [27]. Consequently, the measure of parental alcohol use will, regardless of how the different categories are defined, always contain a non-trivial amount of uncertainty. To validate this study’s results, we will perform a series of sensitivity analyses, using different cut-offs for heavy drinking (presented in the Supplementary Material). The rest were categorised as moderate drinkers (i.e., those who drank at least monthly but at most weekly *or* 2–4 times a week but less than 3 glasses per occasion). These questions about alcohol use have been employed in prior studies, separately and combined, to assess drinking patterns among parents, young adults, and older adults [28–30]. For adolescents with answers from two parents, parental alcohol use was based on the respondent that reported the most frequent alcohol consumption. Thus, children described as having heavy-drinking parents in the text should be interpreted as having at least one parent categorised as a heavy drinker. Those with moderate-drinking parents were used as the reference category in the analyses since we were interested in the difference in health between children with heavy-drinking parents and children whose parents have more typical drinking habits. It is also the largest category and the one that is easiest to interpret. However, we also performed additional analyses changing the reference category to children with heavy-drinking parents.

Adolescents’ gender and age, as well as family structure, household social class, household cash margin, parental level of education, and parental unemployment were included as control variables, based on information in LNU and Partner-LNU. Family structure distinguished between adolescents living in households with two custodial parents, one custodial parent, or in reconstituted families (i.e. one custodial parent and one step-parent or with two foster parents). Household social class was based on parents’ occupations and classified into four categories: manual workers, farmers and entrepreneurs, intermediate/lower non-manual workers, and higher non-manual workers. For those with information from two parents, the classification was based on the principle of dominance order presented by Erikson [31]. Lack of cash margin in the household was defined as at least one parent reporting not being able to come up with 14,000 SEK (∼ 1350 US$ or 1300 €) in a week if needed to. Parental level of education was categorised as ‘compulsory or vocational’, ‘secondary or lower tertiary’, and ‘university degree’, and was based on the parent with the highest level of educational attainment in the household. Parental unemployment was defined as at least one parent in the household being currently unemployed.

### Statistical method

First, the associations between parental alcohol use and all control variables were examined through bivariate cross-tabulations with chi2-tests. Next, linear and binary logistic regression analyses were performed with psychological and somatic complaints and perceived stress as the dependent variables. For each outcome, crude analyses (including one independent/control variable at a time) and an adjusted model (mutually adjusting for all independent/control variables) were conducted. To account for the fact that some adolescents lived in the same households (being siblings or step-siblings), robust standard errors were estimated, clustering at the household level. Regression coefficients (*b)* and odds ratios (OR) with 95% confidence intervals (CI) are presented. All statistical analyses were carried out in Stata version 16.1.

## Results

Descriptive statistics for the total study sample of 909 adolescents are presented in Table 1. The mean values for psychological and somatic complaints were 5.38 and 6.10, respectively. Perceived stress was reported by 20.9% of the sample. Regarding parental alcohol use, 9.4% of the adolescents had abstaining parents, 10.6% had low-consuming parents, 67.4% had moderate-drinking parents, and 12.7% had heavy-drinking parents. The study sample had an equal gender distribution. 69.8% lived with two custodial parents, 15.3% with one custodial parent, and 15.0% in a reconstituted family. 26.5% had parents with manual occupations, 9.8% had parents who were self-employed/farmers, 31.0% had parents who were intermediate or lower non-manual workers, and 32.7% had parents with higher non-manual occupations. Furthermore, 33.4% had parents whose highest level of education was compulsory or vocational school, 38.8% secondary school or lower tertiary education, and 27.7% had at least one parent with a university degree. 14.2% lived in a household where at least one parent lacked a cash margin and 7.4% had at least one unemployed parent. The mean age was 14.2 years.

**Table 1:** Descriptive statistics of the sample, n = 909

	Mean	s.d.	Min	Max
**Psychological complaints**	5.38	1.8	3	12
**Somatic complaints**	6.10	2.2	3	14
**Age**	14.2	2.6	10	18
	n	%		
**Perceived stress**				
No	719	79.1		
Yes	190	20.9		
**Parental alcohol use**				
Abstainers	85	9.4		
Low consumers	96	10.6		
Moderate drinkers	613	67.4		
Heavy drinkers	115	12.7		
**Gender**				
Girls	455	50.1		
Boys	454	49.9		
**Family structure**				
Two parents	634	69.8		
One parent	139	15.3		
Reconstituted	136	15.0		
**Household social class**				
Manual	241	26.5		
Farmers & self-employed	89	9.8		
Intermediate/lower non-manual	282	31.0		
Higher non-manual	297	32.7		
**Household cash margin**				
Yes	780	85.8		
No	129	14.2		
**Parental education**				
Compulsory or vocational	304	33.4		
Secondary or lower tertiary	353	38.8		
University degree	252	27.7		
**Parental unemployment**				
No	842	92.6		
Yes	67	7.4		

Next, we performed crosstabulations with chi2-tests to examine associations between parental alcohol use and sociodemographic characteristics, with results presented in Table 2. Age was trichotomised in this analysis. No statistically significant differences were seen for gender or age. Having heavy-drinking parents was however more common among adolescents living in more socioeconomically advantaged households (i.e., where the parents had non-manual occupations, had a cash margin, were higher educated, and were not unemployed) and among those living with two custodial parents or in reconstituted families. Low-consuming and abstaining parents were, in turn, more common among adolescents living in less socioeconomically advantaged households and among those living with one custodial parent.

**Table 2:** Proportion of adolescents in each parental alcohol use category by sociodemographic characteristics, with chi2-estimates, n = 909

	Parental alcohol use				χ^2^
	Abstainers	Low consumers	Moderate drinkers	Heavy drinkers	
Gender					
Girls	10.6	11.2	66.2	12.1	
Boys	8.2	9.9	68.7	13.2	2.21
**Age (categorical)**					
10–12 years	9.2	8.5	70.6	11.8	
13–15 years	10.3	12.7	65.4	11.7	
16–18 years	8.6	10.6	66.6	14.3	4.60
**Family structure**					
Two parents	9.9	7.7	68.3	14.0	
One parent	13.0	25.2	55.4	6.5	
Reconstituted	2.9	8.8	75.7	12.5	51.07***
**Household social class**					
Manual	16.2	17.8	57.3	8.7	
Farmers & self-employed	14.6	3.4	68.5	13.5	
Intermediate/lower non-manual	6.0	9.2	73.1	11.7	
Higher non-manual	5.4	8.1	70.0	16.5	53.65***
**Household cash margin**					
Yes	5.0	9.2	72.2	13.6	
No	35.7	18.6	38.8	7.0	142.64***
**Parental education**					
Compulsory or vocational	17.1	14.5	58.6	9.9	
Secondary or lower tertiary	5.7	8.2	73.4	12.8	
University degree	5.2	9.1	69.8	15.9	45.70***
**Parental unemployment**					
No	6.5	10.5	70.0	13.1	
Yes	44.8	11.9	35.8	7.5	109.46***

***p < 0.001

Table 3 displays results from linear and binary logistic regressions with psychological complaints, somatic complaints, and perceived stress as dependent variables. In the crude analyses, having heavy-drinking parents, compared with having moderate-drinking parents, was associated with higher levels of psychological complaints (*b* 0.55, p < 0.01) and of somatic complaints (*b* 0.82, p < 0.01), as well as with a higher likelihood of reporting perceived stress (OR 1.62, p < 0.05). These associations were moderately amplified and remained statistically significant in the adjusted models (psychological complaints *b* 0.56, p < 0.01; somatic complaints *b* 0.87, p < 0.001; perceived stress OR 1.67, p < 0.05). Additional analyses revealed that adolescents with heavy-drinking parents also reported higher levels of psychological complaints (p < 0.05) and somatic complaints (p < 0.01) compared with those who had abstaining parents (not shown in table). Compared with those whose parents who were moderate drinkers, adolescents with low-consuming parents reported higher levels of somatic complaints in the crude analysis (*b* 0.65, p < 0.05), but this association was attenuated and turned non-significant in the adjusted model (*b* 0.38, p > 0.05). Having low consuming parents was not associated with psychological complaints or perceived stress, neither in the crude nor in the adjusted analyses.

**Table 3:** Results from linear and logistic regressions with psychological complaints, somatic complaints, and perceived stress as dependent variables, n = 909

	Psychological complaints		Somatic complaints		Perceived stress		
	Crude	Adjusted^a^	Crude	Adjusted^a^	Crude	Adjusted^a^	
	*b* (95% CI)	*b* (95% CI)	*b* (95% CI)	*b* (95% CI)	OR (95% CI)	OR (95% CI)	
**Parental alcohol use**							
Abstainers	0.20 (-0.24, 0.63)	-0.04 (-0.56, 0.47)	0.16 (-0.35, 0.68)	-0.12 (-0.66, 0.43)	1.21 (0.69, 2.10)	1.20 (0.64, 2.24)	
Low consumers	0.40 (-0.08, 0.88)	0.18 (-0.28, 0.63)	0.65* (0.14, 1.16)	0.38 (-0.13, 0.89)	1.17 (0.68, 2.03)	1.00 (0.57, 1.74)	
Moderate drinkers (ref.)	0.00	0.00	0.00	0.00	1.00	1.00	
Heavy drinkers	0.55** (0.18, 0.90)	0.56** (0.21, 0.91)	0.82** (0.35, 1.29)	0.87*** (0.41, 1.33)	1.62* (1.01, 2.60)	1.67* (1.01, 2.75)	
**Gender**							
Girls	0.53*** (0.29, 0.76)	0.49*** (0.25, 0.73)	0.78*** (0.48, 1.07)	0.74*** (0.45, 1.03)	2.40*** (1.72, 3.35)	2.37*** (1.67, 3.36)	
Boys (ref.)	0.00	0.00	0.00	0.00	1.00	1.00	
**Age**	0.07** (0.02, 0.12)	0.07** (0.02, 0.12)	0.04 (-0.02, 0.10)	0.02 (-0.03, 0.08)	1.22*** (1.14, 1.31)	1.23*** (1.14, 1.33)	
**Family structure**							
Two parents (ref.)	0.00	0.00	0.00	0.00	1.00	1.00	
One parent	0.33* (0.00, 0.67)	0.22 (-0.13, 0.57)	0.71** (0.29, 1.12)	0.59** (0.17, 1.01)	1.52 (0.95, 2.42)	1.46 (0.90, 2.39)	
Reconstituted	0.17 (-0.21, 0.56)	0.09 (-0.25, 0.44)	0.24 (-0.16, 0.65)	0.11 (-0.29, 0.50)	1.88** (1.23, 2.88)	1.66* (1.07, 2.59)	
**Household social class**							
Manual	0.37* (0.04, 0.71)	0.50** (0.13, 0.88)	0.43* (0.06, 0.80)	0.25 (-0.20, 0.69)	1.14 (0.75, 1.73)	1.27 (0.77, 2.12)	
Farmers & self-employed	-0.41* (-0.81, -0.02)	-0.24 (-0.62, 0.15)	0.07 (-0.50, 0.63)	0.12 (-0.42, 0.66)	0.87 (0.46, 1.63)	0.94 (0.47, 1.89)	
Intermediate/lower non-manual	0.08 (-0.20, 0.36)	0.23 (-0.07, 0.52)	0.11 (-0.23, 0.45)	0.17 (-0.18, 0.52)	1.07 (0.71, 1.60)	1.17 (0.76, 1.82)	
Higher non-manual (ref.)	0.00	0.00	0.00	0.00	1.00	1.00	
**Household cash margin**							
Yes (ref.)	0.00	0.00	0.00	0.00	1.00	1.00	
No	0.50* (0.05, 0.94)	0.43 (-0.05, 0.92)	0.32 (-0.17, 0.81)	0.15 (-0.39, 0.69)	1.30 (0.82, 2.05)	1.40 (0.82, 2.41)	
**Parental education**							
Compulsory or vocational	-0.06 (-0.36, 0.25)	-0.43* (-0.79, -0.07)	0.38* (0.01, 0.76)	0.17 (-0.29, 0.63)	0.97 (0.64, 1.46)	0.69 (0.41, 1.17)	
Secondary or lower tertiary	-0.06 (-0.38, 0.25)	-0.15 (-0.45, 0.16)	-0.02 (-0.37, 0.32)	-0.06 (-0.42, 0.30)	1.14 (0.76, 1.70)	1.11 (0.72, 1.69)	
University degree (ref.)	0.00	0.00	0.00	0.00	1.00	1.00	
**Parental unemployment**							
No (ref.)	0.00	0.00	0.00	0.00	1.00	1.00	
Yes	0.22 (-0.26, 0.69)	0.11 (-0.42, 0.64)	0.17 (-0.40, 0.75)	0.04 (-0.58, 0.65)	0.90 (0.47, 1.73)	0.87 (0.41, 1.83)	

***p < 0.001 **p < 0.01 *p < 0.05

^a^ Mutually adjusting for all independent/control variables

Turning to the associations between the sociodemographic variables and the health outcomes in the adjusted models; girls reported more psychological and somatic complaints and were more likely to experience perceived stress compared with boys. Older adolescents experienced more psychological complaints and had a higher likelihood of reporting perceived stress. Living with only one parent was associated with somatic complaints and living in a reconstituted family was associated with an increased likelihood of reporting perceived stress. Having parents with manual occupations was associated with psychological complaints. Lastly, having parents with compulsory or vocational education was inversely associated with psychological complaints.

We also performed two types of sensitivity analyses to check the robustness of the results (see Supplementary material). Firstly, a series of linear and binary logistic regressions, similar to those presented in Table 3 but with different cut-offs for heavy drinking, were executed (Tables A1-A4). Overall, these analyses confirmed the associations between having heavy-drinking parents and psychological and somatic complaints. However, the association between parental heavy drinking and perceived stress was consistently weaker compared with the main analysis, and not statistically significant. Furthermore, since parental mental health might be an important confounder in the association between parental drinking and offspring health, we included a variable capturing self-reported nervous trouble, depression, and mental illness from the LNU survey in additional analyses (Table A5). This did not, however, produce any noteworthy changes. Additionally, we performed analyses with paternal and maternal alcohol use separately (Tables A6 & A7).

## Discussion

This study examined associations between parental drinking and offspring subjective health complaints and perceived stress, using information collected separately from parent(s) and adolescents. We distinguished between individuals whose parents were abstainers, low consumers, moderate drinkers, and heavy drinkers. Results from linear and binary logistic regressions showed that having heavy-drinking parents, compared with having moderate-drinking parents, was associated with an increased likelihood of reporting psychological and somatic complaints and perceived stress, even when adjusting for sociodemographic characteristics. These findings reflect earlier studies that have found associations between problematic parental drinking and adverse health among offspring in the general population [11–15] and among adolescents of parents with alcohol use disorder [5–7].

Taking inspiration from prior discussions on adverse outcomes among individuals of parents with alcohol disorder, we propose several underlying mechanisms that may explain our finding that adolescents with heavy-drinking parents were more likely to report subjective health complaints and perceived stress. Parental heavy drinking can negatively affect the parent-child relationship, increase the risk of child abuse or neglect, and contribute to a stressful home environment [5, 8]. Alcohol problems within the family are also connected to feelings of stigma, shame, and guilt among offspring [32], which may directly affect adolescent’s health as well as impair health-promoting activities. Additionally, the risks of experiencing traumatic events or engage in parentification are elevated for offspring in heavy-drinking families, both of which may lead to negative health outcomes [33, 34]. The pathways from parental alcohol use and offspring adverse health could also include more complex mechanisms. Prior studies confirm that there is an increased risk of adolescent drinking among children with heavy-drinking parents [9]. Youth drinking is, in turn, connected to a number of different adverse health outcomes [1]. Living with parents who drink heavily could also negatively affect school performance or reduce contacts with peers among children, both of which may lead to ill health. By taking a life-course approach to harmful alcohol use among parents, consideration has to be made to the risk that children could be prenatally affected by their mother’s heavy drinking, which could have long-lasting health effects [35]. There is also a genetic component that could play an important part, i.e., that parents and children can share a vulnerability to adverse mental health that may lead to a higher consumption of alcohol among parents. All of these pathways could be potential avenues to explore in future studies.

The current study detected no differences in parental drinking by offspring gender or age. However, heavy-drinking parents were more prevalent in more socioeconomically advantaged households and among adolescents living with two custodial parents or in reconstituted families. Contrasting these findings, Pisinger et al. [12] reported that perceived parental alcohol problems were more common among youth not living with both parents and in financially strained households. This discrepancy may be due to Pisinger et al. [12] measuring adolescents’ perception of parental alcohol *problems* and the present study using a measure based on parent-reported frequency and quantity of alcohol consumption. This reasoning may also be interpreted in light of the alcohol harm paradox [36], i.e., that individuals with a lower socioeconomic position tend to drink less but experience more alcohol-related harm. Indeed, a study done on a Swedish adult sample found that education level and earnings were positively associated with higher overall drinking, but negatively associated with binge drinking [37].

### Strengths and limitations

This study benefits from using data based on a nationally representative sample, collected from parents and adolescents separately. It is also one of few studies that have examined associations between parental drinking and offspring health in the general population rather than among individuals of parents with alcohol disorder. Additionally, the data contained reliable measures of control variables, such as on socioeconomic position. Nonetheless, there are some limitations that should be considered. Firstly, due to using cross-sectional data, conclusions regarding causality are difficult to draw. We cannot ascertain that parental drinking patterns preclude offspring subjective health complaints and stress. It is possible that a parent starts to drink heavily *because* of their child’s ill health or externalising behaviour, or a strained parent-child relationship in a reverse causal pathway [38, 39]. In addition, the possibility of omitted variable bias should be acknowledged, i.e., that there may be factors that affect both parental drinking and adolescents’ subjective health complaints and stress, that were not fully captured by our control variables. For example, including a more objective or validated measure of parental mental health than the one used in our sensitivity analyses could potentially affect the results. Further, it is also possible that adolescents’ own drinking is associated with both parental drinking and adolescent health. While investigating the causal chain of events was not within the scope of the current study, forthcoming research should consider including data on offspring alcohol use to facilitate a deeper understanding of the pathways from parental drinking to offspring health.

Secondly, since the data used in the current study are from 2010, it is possible that the results are not valid presently due to changes in, e.g., alcohol habits among parents, family dynamics, or relevant compensating factors. Although some small changes are visible in drinking patterns among Swedish adults since 2010, e.g., total consumption has decreased slightly in most groups younger than 60 years, and the proportion of females who engage in frequent binge drinking has increased [40, 41], the proposed pathways from parental problematic drinking to adolescent health complaints and perceived stress should be as applicable now as they were in 2010 [13] (although very recent data are likely to be affected by changes in society due to the COVID-19 pandemic). Still, analysing the next wave of LNU data when available would, of course, be beneficial to produce more up-to-date knowledge.

Thirdly, the categories of parental alcohol use were not based on any established guidelines or previous studies. In Sweden, heavy drinking has been defined as consuming more than 9 or 14 glasses per week for women and men, respectively. With this definition, about 10% of the adult population are classified as heavy drinkers [40]. Due to differences in operationalisation and data collection, the proportion of youth with heavy-drinking parents varies considerably between studies [42]. In the current study, 12.7% of the adolescents lived with at least one heavy-drinking parent. In a series of sensitivity analyses we used four different cut-offs for heavy drinking compared with the main analysis. These analyses confirmed an association between parental heavy drinking and subjective health complaints among offspring, albeit not as consistently as in the main analyses (and not for perceived stress). Although the proportion of heavy drinkers in the current study seems reasonable in relation to prior studies, our operationalisation of parental heavy drinking should not be read as a recommendation, nor a normative statement of what constitutes high consumption, for future studies or policy makers, but rather it is “heavy” in relation to other study participants. Indeed, prior studies using similar data have differed in their construction of alcohol use measures, indicating that no uniform way of constructing such variables from these items have been established [28–30]. Furthermore, for almost a third of the study sample we only had information from one parent, which might have led to an underestimation of parental alcohol use for these adolescents. Indeed, children were more likely to belong to the abstaining or low-consuming groups if they only had answers from one parent. However, additional analyses showed very similar associations as in the main analysis when only children with answers from two parents were included.

Moreover, recall and/or desirability bias in regards to the items that measure parental drinking might also be present. Apart from the difficulty in remembering consumption levels, revealing one’s alcohol habits could also be shameful. Typically, survey methods in estimating alcohol consumption are associated with underreporting, which in turn, could imply that the levels of consumption presented in the current study are an underestimation [43].

Finally, it should also be acknowledged that even though the Swedish Level-of-Living Survey was based on a nationally representative sample, the attrition in several steps may have compromised the representativeness of the data. The fact that the study was conducted in Sweden, with somewhat lower rates of alcohol consumption than many other European countries [44], also needs to be considered. To be able to make generalisations, additional studies based on data collected in other national settings are needed.

### Future studies

Continued research on alcohol’s harms to others, and especially on adolescents with heavy-drinking parents, is important. Enhanced knowledge about the total societal consequences of alcohol consumption is essential for policy makers to be able to make informed decisions [4, 45]. Studies using longitudinal survey data to examine long-term associations between different levels of parental drinking and offspring outcomes would aid more causal interpretations [29, 46]. Investigating outcomes in other areas besides health, such as relationships with peers or academic achievements, is also a possible avenue for future research.

## Conclusion

Alcohol’s harms to others is gaining more recognition and is important to consider for public health advocates. This study demonstrated associations between parental heavy drinking and adolescent subjective health complaints and perceived stress. Adolescents living with heavy-drinking parents constitute a vulnerable group worthy of adequate public health responses. Implementing policies aiming to support these individuals may be beneficial.

## Electronic supplementary material

Below is the link to the electronic supplementary material.

Supplementary Material 1

## Data Availability

The data that support the findings of this study are available from the Swedish Institute for Social Research [SOFI] (https://www.sofi.su.se/english/2.17851/research/three-research-units/lnu-level-of-living/apply-for-lnu-data) but restrictions apply to the availability of these data, which were used under license for the current study, and so are not publicly available. Data are however available from the corresponding author upon reasonable request and with permission of SOFI.

## Abbreviations

LNU: Swedish Level-of Living survey
OR: Odds ratio
CI: Confidence interval
