# Supplementary material for: Parents’ drinking, childhood hangover? Parental alcohol use, subjective health complaints and perceived stress among Swedish adolescents aged 10–18 years
Source: BMC Public Health. 2023 Jan 24;23:162. doi: 10.1186/s12889-023-15097-w (PMC9872733; doi:10.1186/s12889-023-15097-w)
Supplement: Supplementary file 1 — Supplementary Material 1 [file 12889_2023_15097_MOESM1_ESM.docx]

# Supplementary material

**Table A1.** Results from linear and logistic regressions with psychological complaints, somatic complaints, and perceived stress as dependent variables. Heavy drinkers defined as either drinking daily or 2-4 times a week and at least 4 glasses on average, n=909.

|  | Psychological complaints | Somatic complaints | Perceived stress |
| --- | --- | --- | --- |
|  | Adjusted^a^ | Adjusted^a^ | Adjusted^a^ |
|  | *b* (95% CI) | *b* (95% CI) | OR (95% CI) |
| **Parental alcohol use** |  |  |  |
| Abstainers (9.4%) | -0.08 (-0.59, 0.43) | -0.19 (-0.74, 0.36) | 1.13 (0.61, 2.09) |
| Low consumers (10.6%) | 0.14 (-0.31, 0.60) | 0.31 (-0.20, 0.82) | 0.94 (0.54, 1.64) |
| Moderate drinkers (75.0%) (ref.) | 1.00 | 1.00 | 1.00 |
| Heavy drinkers (5.1%) | 0.68* (0.08, 1.29) | 0.78 (-0.06, 1.63) | 1.39 (0.67, 2.90) |

*p<0.05 ^a^ Adjusted for gender, age, family structure, household social class, parental education, household cash margin and parents’ unemployment

**Table A2.** Results from linear and logistic regressions with psychological complaints, somatic complaints, and perceived stress as dependent variables. Heavy drinkers defined as either drinking daily or 2-4 times a week and at least 2 glasses on average, n=909.

|  | Psychological complaints | Somatic complaints | Perceived stress |
| --- | --- | --- | --- |
|  | Adjusted^a^ | Adjusted^a^ | Adjusted^a^ |
|  | *b* (95% CI) | *b* (95% CI) | OR (95% CI) |
| **Parental alcohol use** |  |  |  |
| Abstainers (9.4%) | -0.06 (-0.58, 0.45) | -0.10 (-0.65, 0.46) | 1.19 (0.64, 2.24) |
| Low consumers (10.6%) | 0.16 (-0.30, 0.62) | 0.40 (-0.11, 0.91) | 0.99 (0.56, 1.74) |
| Moderate drinkers (55.6%) (ref.) | 1.00 | 1.00 | 1.00 |
| Heavy drinkers (24.5%) | 0.25 (-0.04, 0.54) | 0.59** (0.25, 0.92) | 1.34 (0.89, 2.04) |

**p<0.01 ^a^ Adjusted for gender, age, family structure, household social class, parental education, household cash margin and parents’ unemployment

**Table A3.** Results from linear and logistic regressions with psychological complaints, somatic complaints, and perceived stress as dependent variables. In addition to the heavy drinkers from the main analysis, mothers who drink 2-4 times a week and at least 2 glasses on average are also classified as heavy drinkers, n=909.

|  | Psychological complaints | Somatic complaints | Perceived stress |
| --- | --- | --- | --- |
|  | Adjusted^a^ | Adjusted^a^ | Adjusted^a^ |
|  | *b* (95% CI) | *b* (95% CI) | OR (95% CI) |
| **Parental alcohol use** |  |  |  |
| Abstainers (9.4%) | -0.09 (-0.61, 0.42) | -0.13 (-0.69, 0.42) | 1.17 (0.63, 2.18) |
| Low consumers (10.6%) | 0.13 (-0.33, 0.59) | 0.37 (-0.14, 0.88) | 0.97 (0.56, 1.70) |
| Moderate drinkers (59.6%) (ref.) | 1.00 | 1.00 | 1.00 |
| Heavy drinkers (20.5%) | 0.17 (-0.12, 0.46) | 0.56** (0.19, 0.92) | 1.32 (0.85, 2.06) |

**p<0.01 ^a^ Adjusted for gender, age, family structure, household social class, parental education, household cash margin and parents’ unemployment

**Table A4.** Results from linear and logistic regressions with psychological complaints, somatic complaints, and perceived stress as dependent variables. Those who drink 5 glasses or more per occasion also defined as heavy drinkers, n=909.

|  | Psychological complaints | Somatic complaints | Perceived stress |
| --- | --- | --- | --- |
|  | Adjusted^a^ | Adjusted^a^ | Adjusted^a^ |
|  | *b* (95% CI) | *b* (95% CI) | OR (95% CI) |
| **Parental alcohol use** |  |  |  |
| Abstainers (9.4%) | -0.04 (-0.55, 0.48) | -0.06 (-0.61, 0.50) | 1.22 (0.65, 2.28) |
| Low consumers (9.0%) | 0.12 (-0.38, 0.61) | 0.58* (0.02, 1.14) | 0.95 (0.52, 1.74) |
| Moderate drinkers (60.1%) (ref.) | 1.00 | 1.00 | 1.00 |
| Heavy drinkers (20.7%) | 0.39** (0.10, 0.67) | 0.61** (0.24, 0.98) | 1.46 (0.96, 2.21) |

**p<0.01 *p<0.05 ^a^ Adjusted for gender, age, family structure, household social class, parental education, household cash margin and parents’ unemployment

**Table A5.** Results from linear and logistic regressions with psychological complaints, somatic complaints, and perceived stress as dependent variables, n=908-909.

|  | Psychological complaints | Somatic complaints | Perceived stressed |
| --- | --- | --- | --- |
|  | Adjusted^a^ | Adjusted^a^ | Adjusted^a^ |
|  | *b* (95% CI) | *b* (95% CI) | OR (95% CI) |
| **Parental alcohol use** |  |  |  |
| Abstainers | 0.06 (-0.04, 0.16) | 0.03 (-0.07, 0.13) | 1.23 (0.65, 2.31) |
| Low consumers | 0.02 (-0.06, 0.10) | 0.10* (0.00, 0.20) | 0.99 (0.57, 1.73) |
| Moderate drinkers (ref.) | 1.00 | 1.00 | 1.00 |
| Heavy drinkers | 0.10** (0.03, 0.17) | 0.12** (0.03, 0.20) | 1.66* (1.00, 2.75) |
| **Parental mental health problems** |  |  |  |
| No (85.3%) (ref.) | 1.00 | 1.00 | 1.00 |
| Yes (15.1%) | -0.02 (-0.08, 0.03) | -0.02 (-0.09, 0.05) | 0.77 (0.47, 1.26) |

**p<0.01 *p<0.05 ^a^ Adjusted for gender, age, family structure, household social class, parental education, household cash margin and parents’ unemployment

**Table A6.** Results from linear and logistic regressions with psychological complaints, somatic complaints, and perceived stress as dependent variables, only paternal alcohol use, n=740.

|  | Psychological complaints | Somatic complaints | Perceived stress |
| --- | --- | --- | --- |
|  | Adjusted^a^ | Adjusted^a^ | Adjusted^a^ |
|  | *b* (95% CI) | *b* (95% CI) | OR (95% CI) |
| **Paternal alcohol use** |  |  |  |
| Abstainers (7.7%) | 0.16 (-0.40, 0.71) | 0.49 (-0.17, 1.14) | 1.55 (0.80, 3.01) |
| Low consumers (9.5%) | 0.01 (-0.51, 0.53) | -0.02 (-0.55, 0.50) | 0.66 (0.32, 1.39) |
| Moderate drinkers (69.7%) (ref.) | 1.00 | 1.00 | 1.00 |
| Heavy drinkers (13.1%) | 0.62** (0.25, 0.99) | 0.72** (0.21, 1.24) | 1.43 (0.85, 2.41) |

**p<0.01 ^a^ Adjusted for gender, age, family structure, household social class, parental education, household cash margin and parents’ unemployment

**Table A7.** Results from linear and logistic regressions with psychological complaints, somatic complaints, and perceived stress as dependent variables, only maternal alcohol use, original operationalisation, n=802.

|  | Psychological complaints | Somatic complaints | Perceived stress |
| --- | --- | --- | --- |
|  | Adjusted^a^ | Adjusted^a^ | Adjusted^a^ |
|  | *b* (95% CI) | *b* (95% CI) | OR (95% CI) |
| **Maternal alcohol use** |  |  |  |
| Abstainers (15.8%) | -0.17 (-0.60, 0.25) | -0.41 (-0.87, 0.04) | 1.61 (0.92, 2.81) |
| Low consumers (16.5%) | 0.21 (-0.15, 0.56) | 0.25 (-0.18, 0.69) | 1.77* (1.08, 2.90) |
| Moderate drinkers (63.6%) (ref.) | 1.00 | 1.00 | 1.00 |
| Heavy drinkers (4.1%) | 0.56 (-0.07, 1.18) | 1.51** (0.66, 2.36) | 2.28 (0.95, 5.48) |

**p<0.01 *p<0.05 ^a^ Adjusted for gender, age, family structure, household social class, parental education, household cash margin and parents’ unemployment
